# Segmental analysis of cardiac metabolism by hyperpolarized [1-13C] pyruvate: an in-vivo 3D MRI study in pigs

**DOI:** 10.1186/1532-429X-14-S1-P56

**Published:** 2012-02-01

**Authors:** Vincenzo Positano, Maria Filomena Santarelli, Francesca Frijia, Giovanni Aquaro, Luca Menichetti, Vincenzo Lionetti, Giacomo Bianchi, Alessandra Flori, Jan Henrik Ardenkjaer-Larsen, Florian Wiesinger, Rolf F Schulte, Giulio Giovannetti, Fabio A Recchia, Luigi Landini, Massimo Lombardi

**Affiliations:** 1 Fondazione G.Monasterio Regione Toscana-CNR, Pisa, Italy; 2 CNR Institute of Clinical Physiology, Pisa, Italy; 3 Scuola Superiore Sant'Anna, Pisa, Italy; 4 GE Healthcare, Hillerød, Denmark; 5 GE Global Research, Munich, Germany; 6 University of Pisa, Pisa, Italy

## Summary

An image analysis method was developed to obtain a 3D map of the pyruvate metabolites distribution in the LV in hyperpolarised 13C MRI. The obtained polar maps follow the standardized LV AHA segmentation, allowing reproducible and standardized LV segmental analysis.

## Background

MRI with hyperpolarised 13C represents a promising modality for dynamic in vivo spectroscopy and could provide a unique opportunity for non invasive assessment of regional cardiac metabolism [1,2]. The aim of this work is to develop a procedure able to map the metabolites distribution on a standardized 16-segments LV model [3].

## Methods

**Figure 1:**
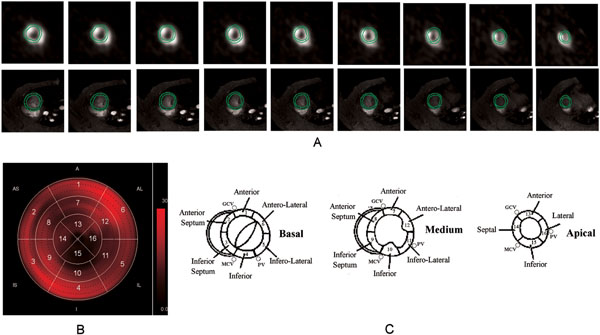
Image analysis procedure: myocardium segmentation (A), resulting metabolite polar map (B), AHA reference model (C).

Seven male pigs (38±2 kg) were imaged on a 3T MRI scanner (GE Excite Hdx) with a 13C birdcage coil. 13-C-pyruvate (20mL of 230mM) was hyperpolarized using a Dynamic Nuclear Polarization technique (Hypersense) and injected in pigs at rest and during occlusion of the left anterior descending coronary artery. Anatomical reference images were acquired by a standard SSFP sequence. Metabolic information were obtained using a volumetric IDEAL spiral CSI sequence [4] prescribed on the same region imaged by the reference anatomical sequence (FOV 30x30x10cm, TE=0.9ms, FA=7°), after 20 sec from the injection starting. 3D axial volumes were reconstructed for Pyruvate, Lactate, and Bicarbonate metabolites. Short axis (SA) cardiac views were reformatted by PMOD software by using the anatomical images as reference. Quantitative analysis was performed on SA views by custom (HIPPO-C13) software developed in IDL 8.0. Myocardial contours were manually defined in anatomical SA views covering the LV (Fig 1.A) A reference point corresponding to superior insertion of the right ventricle wall was defined. Slices were classified as basal, middle, or apical by the operator. Starting from the reference point, myocardium was divided in six (basal and medium slices) or four (apical slices) equiangular segments. Corresponding polar maps were extracted as illustrated in Fig. 1.B. Values of metabolites signal in corresponding segment of basal, middle, and apical slices were averaged leading to a 16-segments model of metabolite distribution, following AHA guidelines [3] (Fig 1.C).

## Results

**Figure 2:**
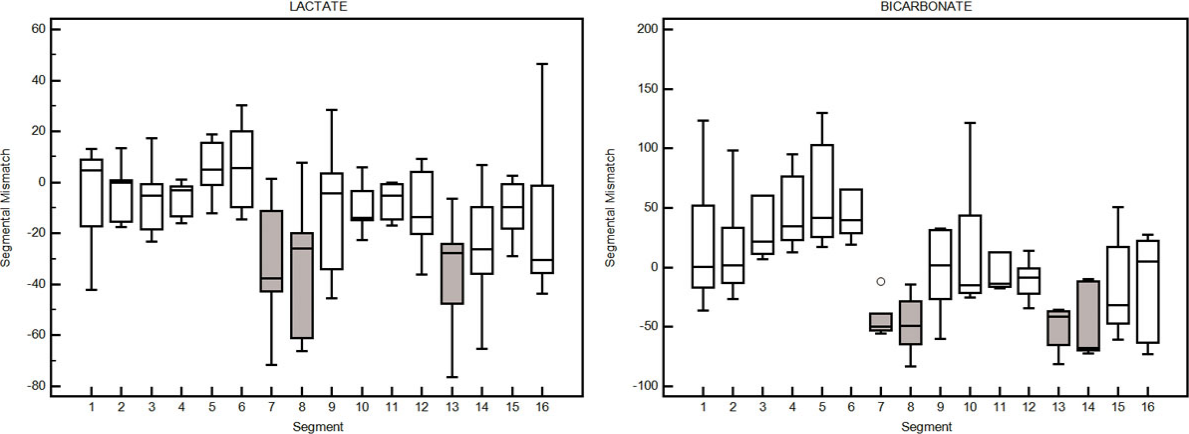
Segmental mismatch between basal and occlusion condition for lactate and bicarbonate. Gray blocks indicate statistically significant difference between the two conditions.

Differences in metabolite signal among acquisitions were normalized by assessing segmental variation as percent difference between segmental values and global LV value. To assess the difference between basal and occlusion conditions, the mismatch between two segmental variation maps was defined as percent difference. Fig. 2 shows the segmental mismatch for lactate and bicarbonate distribution. ANOVA analysis revealed a significant (p<0.001) influence of segment location on mismatch. The most affected segments (grey) correlate well with the occlusion location.

## Conclusions

MRI with hyperpolarised 13C allows mapping the pyruvate metabolites distribution in the LV following the standardized AHA segmentation. Macroscopic changes in metabolites concentration due to coronary occlusion are consistently detected. Further studies are needed to characterize the sensitivity of the method to fine variations of regional metabolite concentration.

## Funding

No specific funding was received for this study.
